# Immunoprecipitation and mass spectrometry define TET1 interactome during oligodendrocyte differentiation

**DOI:** 10.1186/s13578-020-00473-5

**Published:** 2020-09-16

**Authors:** Ming Zhang, Kaixiang Zhang, Jian Wang, Yuming Liu, Guangxin Liu, Weilin Jin, Shengxi Wu, Xianghui Zhao

**Affiliations:** 1 Department of Neurobiology, School of Basic Medicine, Fourth Military Medical University, Xi’an, 710032 Shaanxi China; 2 School of Electronic, Information and Electrical Engineering, Shanghai Jiao Tong University, Shanghai, 200240 China

**Keywords:** Oligodendrocyte, TET1, DNA dioxygenase, Mass spectrometry, Co-immunoprecipitation

## Abstract

Ten-eleven translocation (TET) proteins, encoding dioxygenase for DNA hydroxymethylation, are important players in nervous system development and disease. In addition to their proverbial enzymatic role, TET proteins also possess non-enzymatic activity and function in multiple protein–protein interaction networks, which remains largely unknown during oligodendrocyte differentiation. To identify partners of TET1 in the myelinating cells, we performed proteome-wide analysis using co-immunoprecipitation coupled to mass spectrometry (IP-MS) in purified oligodendrocyte precursor cells (OPCs) and mature oligodendrocytes (mOLs), respectively. Following a stringent selection of MS data based on identification reliability and protein enrichment, we identified a core set of 1211 partners that specifically interact with TET1 within OPCs and OLs. Analysis of the biological process and pathways associated with TET1-interacting proteins indicates a significant enrichment of proteins involved in regulation of cellular protein localization, cofactor metabolic process and regulation of catabolic process, et al. We further validated TET1 interactions with selected partners. Overall, this comprehensive analysis of the endogenous TET1 interactome during oligodendrocyte differentiation suggest its novel mechanism in regulating oligodendrocyte homeostasis and provide comprehensive insight into the molecular pathways associated with TET1.

## Introduction

Emerging as new epigenetic factors for neural development, TET family members have been associated with the process of oligodendrocyte (OL) differentiation [1]. Axon myelination by OLs enables saltatory conduction of action potentials and provides long-term trophic support for axons, maintaining integrity throughout the central nervous system (CNS) [2]. TET proteins were initially identified as dioxygenase for DNA hydroxymethylation and oxidize 5-methylcytosin (5mC) to 5-hydroxymethylcytosine (5hmC) to initiate the DNA demethylation process. Three members of the mammalian TET gene family have been defined, TET1, 2 and 3, and all TET proteins contain a similar C-terminal catalytic domain, which confers α-ketoglutarate (α-KG)-and iron (II)-dependent dioxygenase activity [3].

Extensive studies have focused on the catalytic enzymatic roles of TET family proteins in regulating various process from development to disease in a cell-type and context-dependent manner [4]; while some investigations illustrated a non-catalytic activity of TET proteins. For instance, several studies have reported that TET proteins can interact with other epigenetic modifiers or transcriptional regulators independent of their enzymatic activity [5–9], such as histone deacetylase 2 (HDAC2), O-GlcNAC transferase (OGT), Sin3A complex and hypoxia inducible factors (HIFs). Furthermore, Cartron et al. observed that TET1 interacts with MeCP2, HDAC1/6/7, EZH2, mSin3A, PCNA, and LSD1 to control its DNA-demethylating function [10]. Thus, TET family proteins may work as transcriptional activator or repressor through their enzymatic and non-enzymatic activity in multiple cellular processes.

In previous study, we have identified that TET1 is highly expressed in oligodendrocytes and siRNA mediated TET1 silencing impairs OL differentiation [1]. Here, to gain insights into the interacting patterners for TET1 in oligodendrocytes, we used a mass spectrometry (MS)-based proteomics approach to characterize potential interactomes in this glia type. Knowledge of these new TET1-interacting proteins could provide valuable resources for understanding the mechanism of this epigenetic regulator in oligodendrocyte biology.

## Materials and methods

### Oligodendrocyte primary culture and immunostaining

Isolation and culture of mouse OPCs were modified as previously described [11]. Briefly, brains were removed from P2 C57 mouse pups, and the cortices were dissected. Cortical pieces were enzymatically digested followed by mechanical dissociation. Cells were resuspended in DMEM with 10% fetal bovine serum and plated onto 60 mm dishes. When primary mixed glial cultures reached ~ 70% confluent, we substituted FBS with B104-conditioned medium (B104-CM) modified oligodendrocyte growth medium to enrich OPCs. Purified OPCs were prepared by a chemical-based method and were seeded onto poly-l-ornithine–coated 35-mm dishes or coverslips. OPCs were amplified in growth medium (Sato medium supplemented with fibroblast growth factors, and platelet derived growth factor AA) and were initiate to differentiate with differentiation medium (Sato medium supplemented with triiodothyronine, ciliary neurotrophic factor, and N-acetyl-l-cysteine).

For immunocytochemistry assay, OL cultures were fixed in 4% paraformaldehyde. After permeabilization for 15 min, samples were incubated with primary antibody for 1 h at room temperature followed by fluorescent secondary antibody for another hour. Samples were counter-stained with DAPI and visualized with an Olympus confocal microscope. Antibodies against OL markers are rabbit anti-PDGFRa (BD biosciences, 558774) and rat monoclonal anti-MBP (Millipore, MAB386).

### Immunoprecipitation and Western blot assay

Cell cultures were lysed in RIPA buffer (150 mM NaCl, 50 mM Tris–HCl (pH 7.4), 1% NP40, 1 mM PMSF, 1 × Roche complete mini protease inhibitor cocktail, and 1 × Pierce phosphatase-inhibitor cocktail). Protein concentrations in centrifugation-clarified cell lysates were measured by the BCA Protein Assay Kit (Pierce). Co-IP was performed using the Catch and Release v2.0 Reversible Immunoprecipitation System (Millipore, 17–500) according to the manufacturer's instructions. All collected protein complexes were eluted with 10 μl of 5 × loading buffer by boiling for 5 min and the eluates were subjected to SDS-PAGE. Antibodies used for immunoprecipitation were rabbit anti-TET1 (Active Motif, 61443), rabbit anti-Olig2 (Millipore, AB9610), rabbit anti-Olig2 (a gift from Dr Charles Stiles, Harvard University, Cambridge, MA) and rabbit anti-Flag (Proteintech, 20543–1-AP).

For Western blot assay, protein samples from cell lysates or Co-IP elution were extracted using RIPA lysis buffer with the protease inhibitor (Roche). Protein concentrations were measured using BCA protein assay (Thermo scientific ™) according to the manufacturer’s instructions. Equivalent amounts of protein (20 μg) were separated on SDS-PAGE gel and transferred to Hybond PVDF (Roche). For protein blotting, the following primary antibodies were used: rabbit against-β-tubulin (Proteintech, 10068–1-AP), rabbit against-TET1 (Genetex, GTX64332), mouse anti-Olig2 (Millipore, MABN50A4), rabbit against-GFP (Abcam, ab6673). Signals were developed with horseradish peroxidase–conjugated secondary antibodies (Abbkine), followed by ECL kit (Zeta LIFE).

### MS analysis and protein identification

After co-immunoprecipitation, equal amounts of proteins were loaded in 4–12% SDS-PAGE gels and stained with Coomassie blue G250 (BioRad). After staining, the bands higher than 10 kDa were excised into individual fractions, excluding the stained IgG-H (52 kDa). These fractions were then further excised into small pieces and placed into a 1.5-ml tube. Sample preparation used for Q-Exactive mass spectrometry was performed according to the standard protocol [12]. After destaining and shrinking, the gel was treated with 20 mM DTT for protein reduction, followed by 50 mM iodoacetamide (IAA) treatment for alkylation. Protein digestions were performed with trypsin at 37 °C overnight and the digested proteins were then desalted for LC–MS/MS analysis (AB SCIEX TOF/TOF™ 5800 system, USA). Proteins were identified using Protein Pilot 4.0TM software (AB Sciex, USA) [13].

### Bioinformatic analyses

Proteins detected in TET1-IP products but not in IgG-IP products or the ratio of Log_2_ value of LFQ intensity (normalized against IgG) > 2 were identified as TET1-interacting proteins. To identify the biological and functional properties of TET1-interacting proteins, we used Gene Ontology (GO) annotation by searching the GO website (https://www.geneontology.org). The GO and pathway enrichment analysis was performed and visualized online with Metascape (https://metascape.org).

To identify protein–protein interaction network of TET1 partners in OPC and OL, Cytoscape 3.6.1 [14] together with the ClueGo plugin [15] was used for enrichment analysis. GO terms were considered significant at the *p* < 0.01 level. Filtered terms were visualized in a network layout with circular nodes. Color-coded nodes represent different GO terms, and node size correlate with enrichment *p* value. The associated proteins were visualized in the network with small nodes and connected with related GO terms by thick edges. Kappa statistic was used to calculate the overlap of proteins associated with any two GO terms. All the nodes with κ value ≥ 0.4 were connected by edges, with thickness were corresponding to kappa score [16]. All final figures were assembled using Adobe Illustrator CC 2019.

To facilitate comparison of gene expression levels and interactions at different cell stages, proteins specifically interacting with TET1 in OPC/OL were transformed to Gene symbol on UniPort (https://www.uniprot.org). After that, four groups genes were uploaded to explore the intersection among different cell stages by Venny 2.1.0 (https://bioinfogp.cnb.csic.es/tools/venny/index.html). Sets of overlapping genes were visualized in Venn diagrams that were generated in Adobe Illustrator.

The clustered heatmap was constructed to visualize related RNA gene expression, using the HemI 1.0.2 Heatmap Illustrator Toolkit with hierarchical clustering. Log_2_ transformation was applied to the matrix before being visualized. The expression value was presented from 0 (blue) to 8 (violet).

### RNA-Seq data analysis

The transcriptome profiling was from previously published and deposited dataset (GSE66047) [17]. RNA-seq reads were mapped using TopHat2 with settings of “read mismatches = 2” and “read gap length = 2” (https://ccb.jhu.edu/software/tophat/ index.shtml). TopHat output data were then analyzed by DEGseq to compare the changes of gene expression between OPC and OL, based on the calculate RPKM values for known transcripts in mouse genome reference. Volcano Plot of gene differential expression was generated using R Package (https://www.r-project.org).

### Cell line transfection

Hela cell transfection was performed using the DNA Xfect Transfection Reagent (Takara, 631,317) according to the manufacturer’s instructions. Full length TET1 constructs and RFP-Olig2 constructs were obtained from Dr. Heinrich Leonhardt [18]. pEGFP-N1 and pCDNA3.1 plasmid were used for control transfection. Forty-eight hours after transfection, cells were harvested for Co-IP assay as described above.

### Statistical analysis

All data are expressed as the mean ± SEM. Student’s t-test was used to analyze the differences between the means. *p* < 0.05 was considered statistically significant.

## Results

### Analysis of endogenous TET1 interactors in oligodendrocytes by co-immunoprecipitation and mass spectrometry

**Fig. 1:**
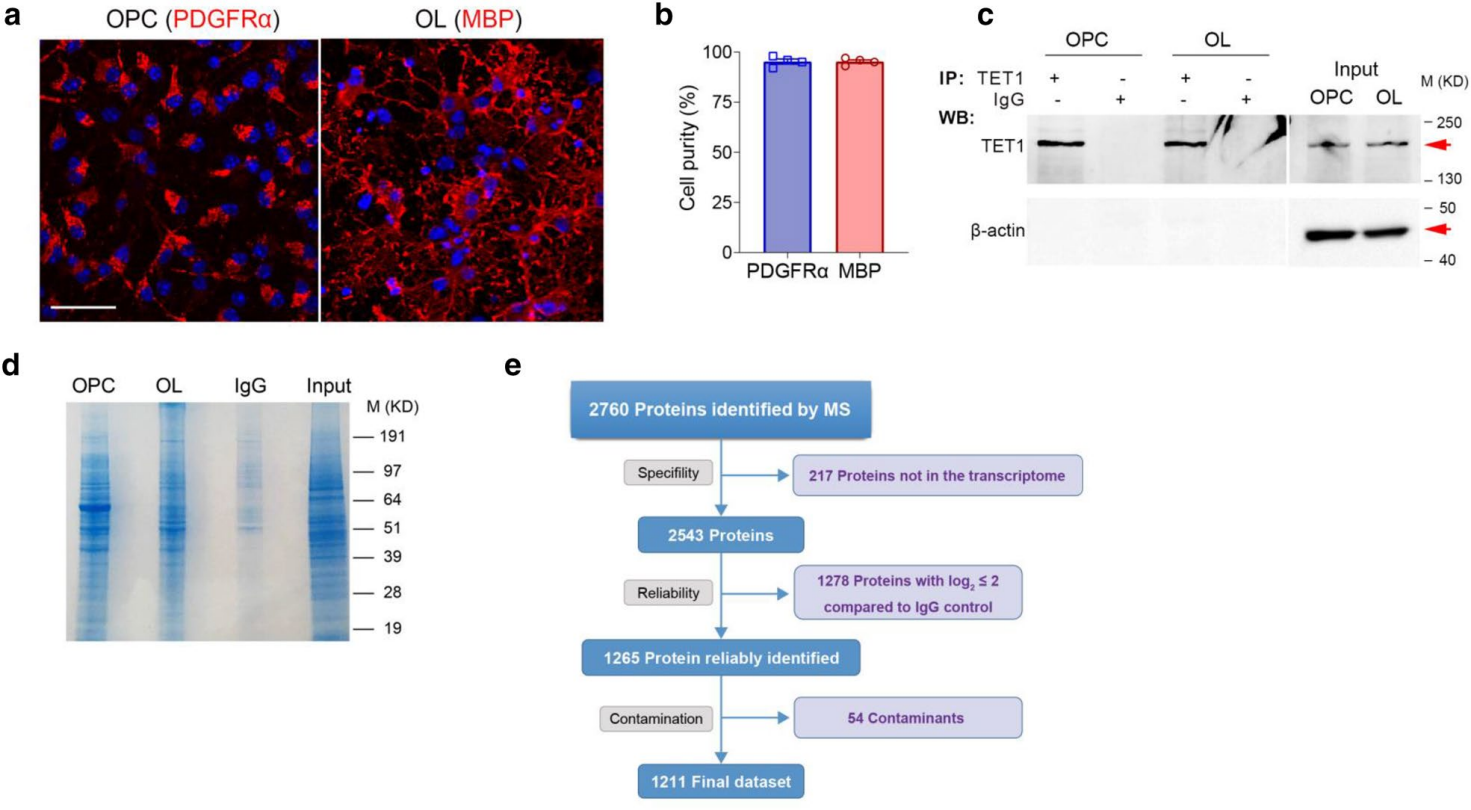
Immunoprecipitation and mass spectrometry revealed endogenous TET1 interactors in oligodendrocytes. **a** Immunostaining with lineage specifc oligodendrocyte makers indicated the purity of oligodendrocyte cultures. Antibody against PDGFRα labeled OPCs and against MBP labeled mature OLs. Scale bar, 50 μm. **b** Quantification the percentage of PDGFRα^+^ and MBP^+^ cells in OPC and OL stage, respectively. **c** Westernblot assay identified TET1 in TET1-IP products in both OPC and OL groups. Red arrows indicate the predicted bands for endogenous TET1. IP input from both groups was used as positive control for TET1. β-actin was used as loading control and negative control. TET1 did not appear in IgG-IP products. **d** Coomassie blue staining of SDS-PAGE for TET1-IP products from OPC and OL samples. **e** Analysis workflow for the filtering of MS data. Specificity, reliablity and contamination filteration result a final dataset of 1211 proteins in OPC and OL samples

To discover TET1 protein partners, we performed proteomic study using co-immunoprecipitation (co-IP) of the endogenous protein in oligodendrocyte precursor cells (OPCs) and mature oligodendrocytes (mOLs), respectively. The purity of cell cultures was accessed by immunostaining with stage specific markers, which revealed > 95% PDGFRα^+^ OPCs and MBP^+^ OLs (Fig. 1a, b). Cell lysates from purified OPCs and OLs were immunoprecipitated with Catch and Release kit. Normal IgG pull-down was included as negative control. Immunoblot analysis of the IP fractions showed that TET1 was enriched in the pulldown products from both groups (Fig. 1c), indicating the successful TET1 immunoprecipitation from cell extracts. Co-immunoprecipitated proteins were resolved in SDS-PAGE 4–12% gradient gel (Fig. 1d) and Coomassie blue staining of the gels identified multiple bands that were not present in IgG controls. Regions with protein bands were cut off and underwent in-gel digestion and Mass Spectrometry (MS) protein identification.

Co-IP experiments were performed twice, and MS identified proteins were combined for analysis. A total of 2760 proteins were identified with Protein Pilot 4.0™ software (Fig. 1e). To exclude the possible non-oligodendrocyte lineage transcripts in MS identified proteins, we performed RNA-seq for purified OPCs and OLs. Samples for each group were in triplicate and 14,795 genes in total showed expression in two stage of oligodendrocytes. In this step, 217 proteins were removed from MS results. Then, by comparing the ratio of Log_2_ value of LFQ intensity to IgG control (> two folds), 1265 proteins were identified to interact with TET1 in OPCs and OLs. Subsequently, to remove potential contaminants, we used the CRAPmoe database [19], which contains a compilation of proteins frequently identified in affinity purification MS control. We considered that any proteins in our data with a CRAPome max spectral count over 200 (1.99% genes in OPC and 1.77% in OL) or the multiplication for the number of experiments and max spectral count over 100 were contaminants [20]. This step identified a total of 54 contaminants. Finally, the filtration step yielded a dataset of total 1211 proteins in OPC and OL precipitates (Fig. 1e).

### TET1 interactomes in OPC and OL are not related to the expression level

**Fig. 2:**
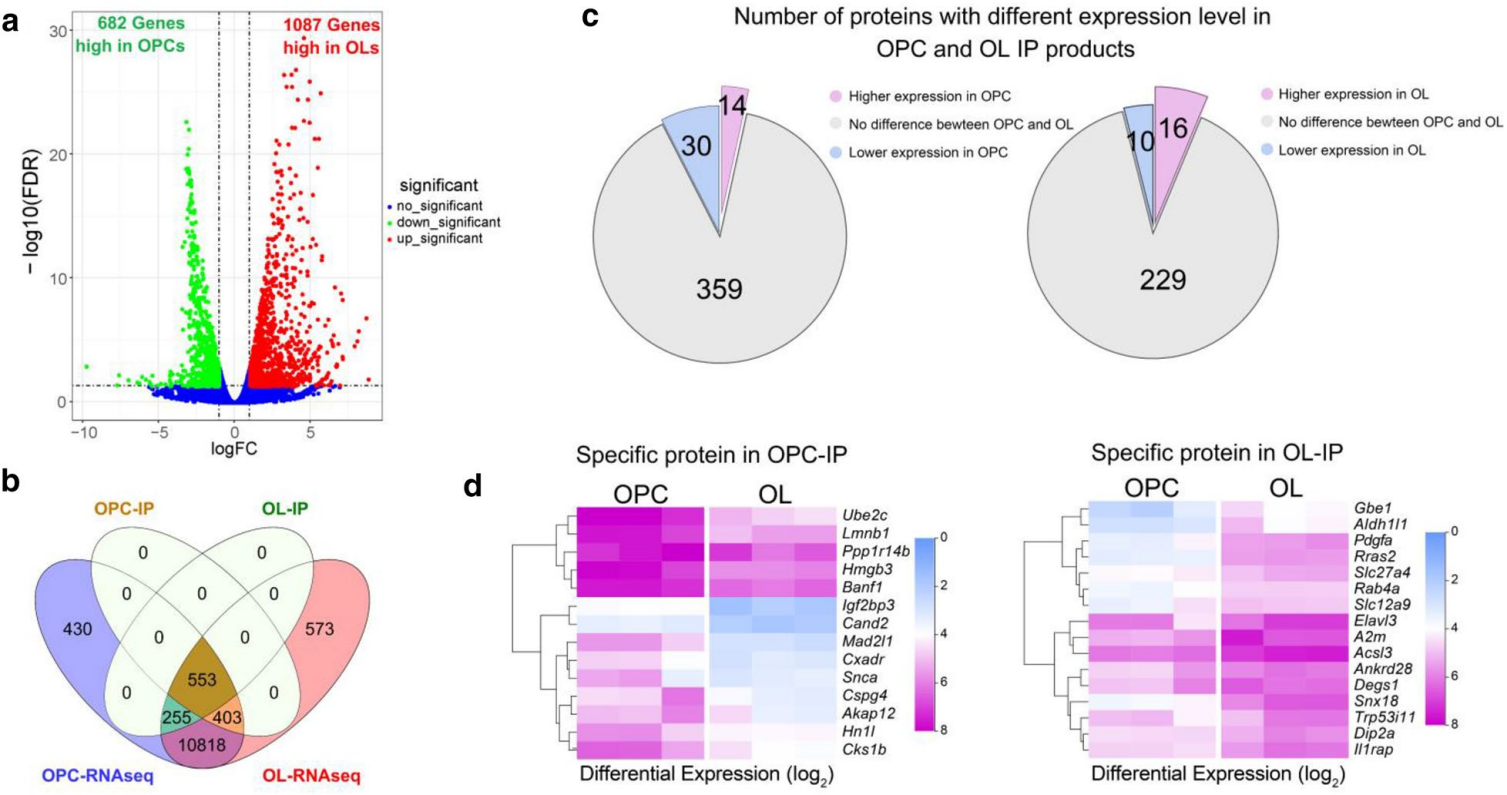
Venn diagram and correlation analysis of identified proteins in MS and RNA-seq. **a** Volcanic plot of the differentially expressed genes in OL compared with OPC [17]. **b** Venn diagram reveals the comparation between TET1 interactome and oligodendrocyte transcriptome at different stages. TET1-IP products in OPC and OL groups were compared with OPC and OL transcriptomes.**c** Pie charts show the number of stage specific TET1 interactors at different expression level. **d** Heatmap reveals hierarchical clustering of TET1 interactors with upregulated expression in OPC or in OL, respectively

To investigate if TET1 interactome is oligodendrocyte stage specific, we compared the dataset with RNA transcriptome for OPC and OL. There were 682 genes showing higher expression level in OPCs and 1087 gene showing higher expression level in OLs (FDR < 0.05, Log_2_ > 1 or < -1) (Fig. 2a) [17]. Comparing TET1 interactome with RNA transcriptome of OPC and OLs, Venn diagram confirmed that all 956 proteins (553 + 403) of OPC-IP group were in the OPC transcriptome and all 808 proteins (553 + 255) of OL-IP group were in OL transcriptome (Fig. 2b). Respectively, 403 and 255 proteins were OPC and OL specific, and 553 proteins were shared between two groups (Fig. 2b). Intriguingly, none of the stage-specific transcripts were TET1-IP products, indicating that TET1 may work as a lineage, rather than a cell stage regulatory protein during oligodendrocyte differentiation.

To further reveal if stage specific TET1 interactors were related to their expression levels, we compared their expression between OPC and OL. We noticed that ~ 89.08% (359 out of 403 proteins) and ~ 89.80% (229 out of 255 proteins) of TET1 interactors in OPC and OL groups, respectively, did not show significant expression priority in either stages (Fig. 2c). Only 14 out of 403 TET1 interactors in OPC group and 16 out of 255 in OL groups were highly expressed in corresponding groups (Fig. 2c). Heatmap revealed the expression of these stage specific TET1 interactors that highly expressed in corresponding stages (Fig. 2d). Together, these observations indicate that most of TET1 partners were not related to their expression levels.

### TET1 interacting proteins participate in novel functions and pathways

**Fig. 3:**
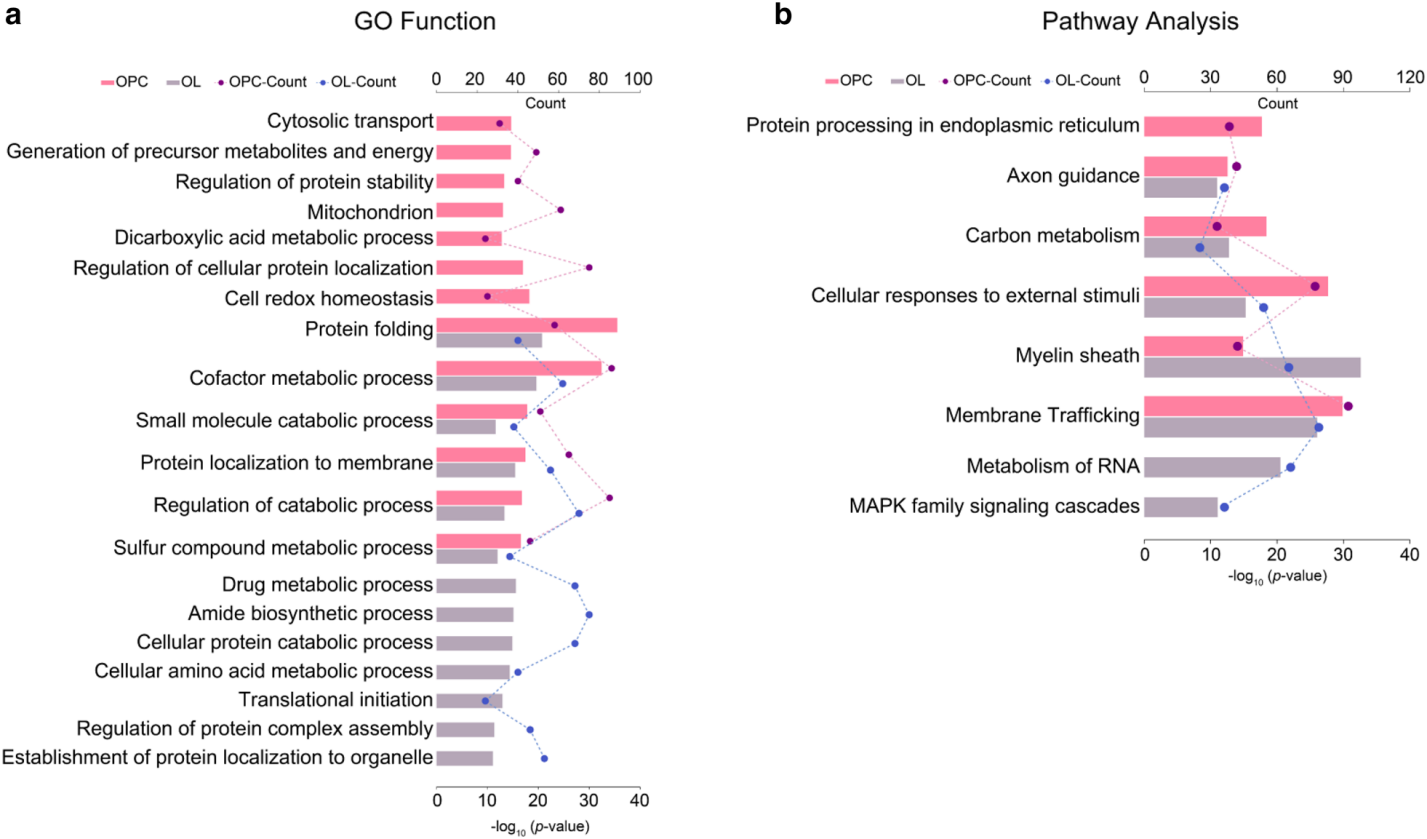
GO and pathway analysis for TET1-interacting proteins in oligodendrocytes. **a** Top 20 Gene ontology annotation terms of TET1 interactors in OPC and OL. Six GO terms are shared between two groups. **b** KEGG pathway and Reactome Gene Sets enrichment analysis reveal stage specific and lineage enriched pathways for TET1 interactors

To illustrated functional association of these TET1 partners, gene ontology (GO), KEGG Pathway and Reactome Gene Sets analysis were performed. Interestingly, numerous TET1-IP partners suggested novel roles for TET1 beyond its DNA dioxygenase activity. Among the top twenty enrichments, gene terms of protein homeostasis relevant process (namely protein folding, protein localization, protein stability, protein complex assembly) and molecular metabolic and catabolic process (including cofactor metabolic process, small molecule catabolic process, regulation of catabolic process, sulfur compound metabolic process) were implicated in both OPC and OL groups (Fig. 3a). Meanwhile, GO terms about mitochondrion organization and cell redox homeostasis were predominant themes for OPCs; and translational initiation was enriched in OLs precipitates (Fig. 3a).

In addition, KEGG pathway and Reactome Gene Sets analysis unveiled an enrichment of proteins functioning in membrane trafficking, cellular responses to external stimuli, myelin sheath, carbon metabolism and axon guidance in TET1-IP products from both OPC and OL (Fig. 3b). Specifically, RNA metabolism and MAPK family signaling cascades were identified in OLs (Fig. 3b). These annotations provide valuable resources for further investigations on elucidating TET1 function in oligodendrocyte lineage.

**Fig. 4:**
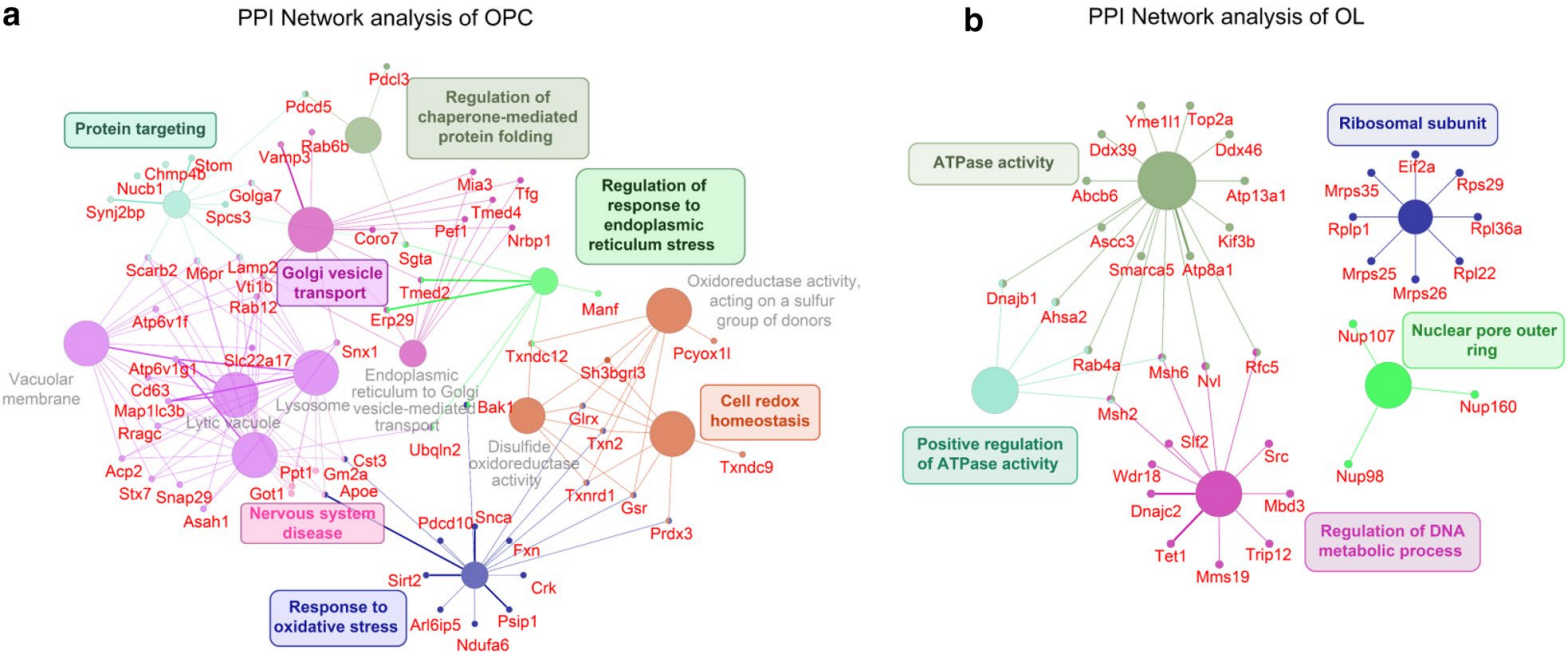
Protein–protein interaction network for the leading terms with associated proteins. Leading terms from CluoGO analysis in OPC (**a**) and OL (**b**) were placed into a separate network. All associated proteins from the list of OPC or OL specific interacting proteins were visualized as nodes and connected to the appropriate term. The small nodes represent proteins, and the thickness of the edges from nodes mark confidence of the interactions. Where a protein was associated with multiple terms, the nodes was connected with multiple edges corresponding to their term colors

To further investigate the interaction network of TET1 partners in OPCs and OLs, Cytoscape with ClueGO and Cluepedia plugin was used. The enriched complexes in each group were labeled in Fig. 4 as clusters of highly interconnected proteins, which revealed distinct interaction network pattern for TET1 in different oligodendrocyte stage. For instance, in OPC protein–protein interaction (PPI) network, proteins involved in Golgi vesicle transport (such as Vamp3, Golga7, Vti1b and Snx1), Cell redox homeostasis (such as Tnx2 and Gsr), nervous system disease (such as Got1, Gm2a, Apoe and Cst3) and other functions were identified (Fig. 4b). In the networks of OL, functional enrichment terms including ATPase activity (such as Atp8a1, Smarca5, and Dnajb1), regulation of DNA metabolic process (Dnajc2, Wdr18 and Slf2), ribosomal subunit (Rplp1, Eif2a and Rps29) and nuclear pore outer ring (Nup107, Nup160 and Nup98) were revealed (Fig. 4b). These analyses suggest that TET1 can work in different interaction networks in OPC and OL, which is more complex in OPC stage.

### Transcription factors, especially HDAC1 and Olig2, are stage specific TET1 binding factors in oligodendrocytes

**Fig. 5:**
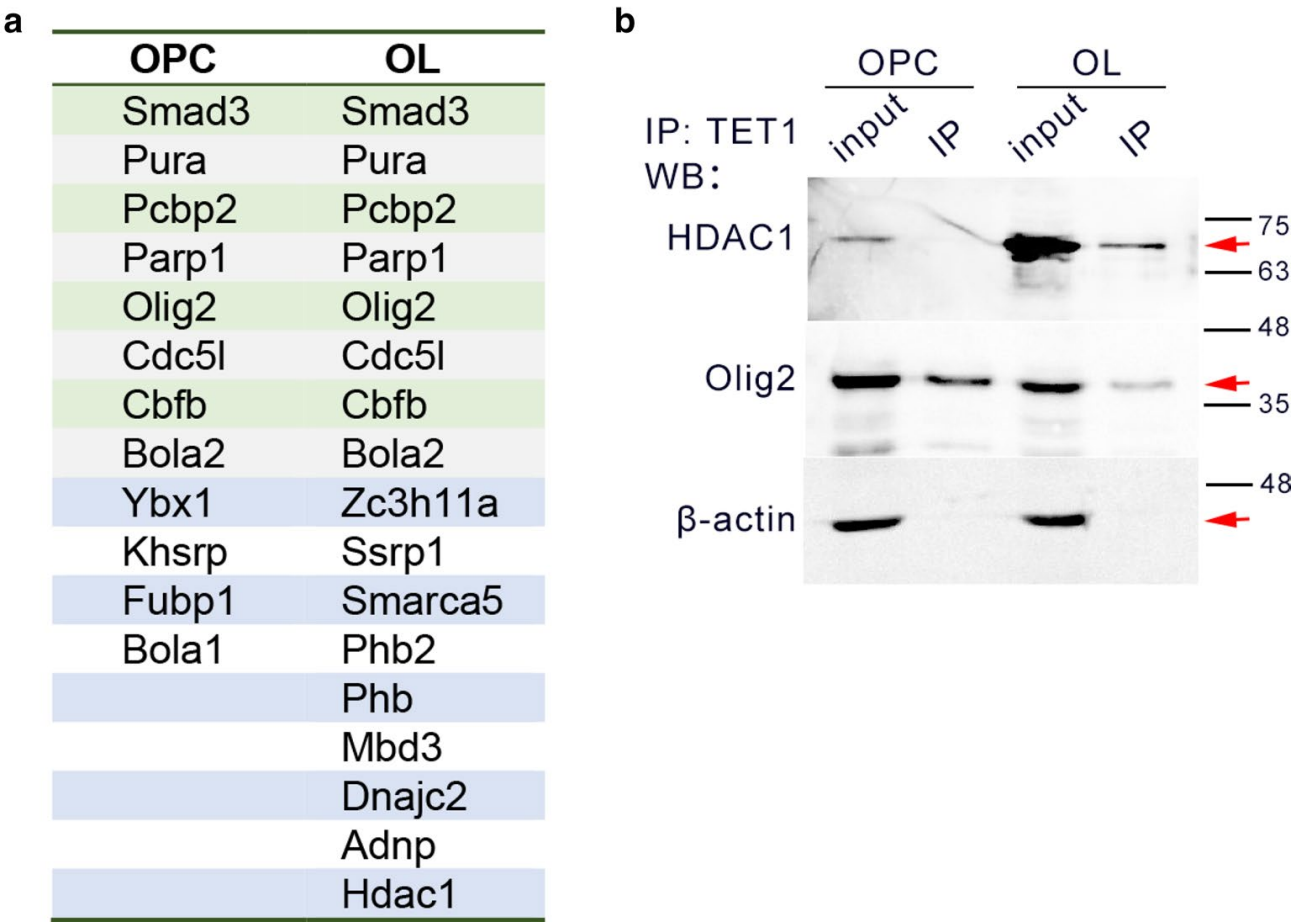
Identification of transcription factors as TET1 partners during oligodendrocyte differentiation. **a** Summary of transcription factors showing interaction with TET1 in OPC and OLs.** b** Westernblot assay confirm the interaction of TET1 and HDAC1 or Olig2 in OPCs and OLs. Red arrows indicate the predicted bands for endogenous TET1 and Olig2. IP input from both groups was used as positive control for TET1. β-actin was used as negative control and loading control. TET1 did not appear in IgG-IP products

Except for DNA demethylation, TET1 also works in various ways and crosstalks with partners to regulate gene expression [21–23]. Then we searched for transcription factors (TFs) in TET1 interactome with TcoF-DB v2 database. We found that 11 out of 956 partners in OPCs and 17 out of 808 partners in OLs were TFs (Fig. 5a). Several TFs were appeared in both stages, and others showed stage specific interaction with TET1. This observation suggests the possible involvement of TET1 in multiple and different transcription factor complex during oligodendrocyte differentiation.

To validate certain TFs as TET1 partners in oligodendrocytes, we first performed Western blots for IP products from OPCs and OLs. Olig2 is a lineage specific TF that modulates the progression of OL development [24]. Another TET1 partner, histone deacetylases 1 (HDAC1), is an important component of epigenetic modification in regulating OL differentiation [25]. We noticed that HDAC1 appeared exclusively in MS results from OLs, but Olig2 binds to TET1 in both OPCs and OLs (Fig. 5a). Western blot assay using Olig2 and HDAC1 antibodies confirmed the existence of Olig2 and HDAC1 in TET1-IP products from OPCs and OLs (Fig. 5b), which was in consistent with MS results.

**Fig. 6:**
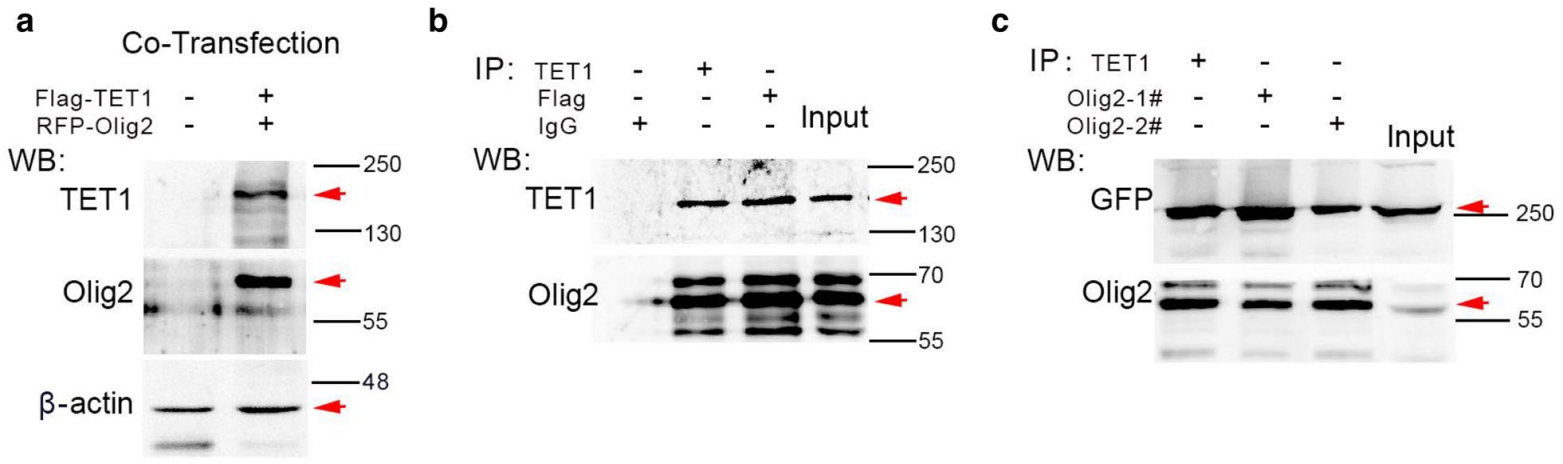
Interaction of TET1 and Olig2 in Hela cells overexpressing these proteins. **a** Western blot assay confirm the overexpression of TET1 and Olig2 in Hela cells. Red arrows indicate the predicted bands for Flag-TET1 and RFP-Olig2.** b** Immunoprecipitation with TET1 or Flag antibodies reveal the interaction between TET1 and Olig2 in Hela cells. Red arrows indicate the predicted bands for Flag-TET1 and RFP-Olig2.** c** Reciprocal co-IP assay with Olig2 antibodies confirm the interaction between TET1 and Olig2 in Hela cells. Red arrows indicate the predicted bands for Flag-TET1 and RFP-Olig2

To further confirm the interaction between Olig2 and TET1, we then tested their binding in transfected cell lines. Flag-TET1 and RFP-Olig2 plasmids were co-transfected into Hela cells and overexpression of these two proteins were identified in Western blot assay (Fig. 6a). Co-IP was then performed in whole cell extracts with antibody against TET1 or Flag, and matched normal IgG as negative control. Immunoblot with antibody against Olig2 detected protein bands with predicated molecular size, indicating the interaction between TET1 and Olig2 (Fig. 6b).

Next, we performed a reciprocal co-IP assay for TET1 and Olig2. Two antibodies against Olig2 were used for IP and another TET1 plasmid, GFP-TET1, was used for co-transfection in Hela cells. Western blot assay with GFP antibody showed strong TET1 signals with predicated size in both Olig2 antibody IP products (Fig. 6c). Taken together, these data provide evidence for the validity of IP-MS results and confirm specific interactions of selected candidate proteins with TET1 within OL lineage.

In conclusion, we identified a group of proteins as TET1-interacting partners in oligodendrocytes after eliminating nonspecific binders. In-depth bioinformatics analysis clarifies previously unknown molecular functions of TET1 in protein folding, cofactor metabolic process, small molecule catabolic process, regulation of catabolic process, sulfur compound metabolic process and protein localization to membrane. Additionally, we identified and validated transcriptional factors regulating OL differentiation as TET1-interacting partners, especially Olig2. Knowledge of these new TET1-interacting proteins and PPI networks could provide valuable resources for understanding the functions of this family in oligodendrocyte biology.

## Discussion

We used IP-MS to identify TET1 interacting proteins and gain insight into the biological functions of this protein in oligodendrocytes. Stringent filtration steps including comparation with oligodendrocytes transcriptome and with CRAPome database were applied to exclude non OL lineage proteins and potential contaminant proteins during IP-MS. Taking the list of TET1 interactors in OPCs and OLs, we next used GO and pathway analysis to link TET1 interacting proteins to putative biological functions and pathways, which revealed that TET1 may be involved in protein homeostasis (protein localization, protein stability and assembly), myelin sheath and molecular metabolic and catabolic process. Notably, the results revealed gene enrichment for mitochondrion organization, nucleotide binding and cell redox homeostasis in OPCs, and translational initiation, RNA metabolism, and MAPK family signaling cascades in OLs.

Further analysis of the protein–protein interaction (PPI) networks for TET1 showed that in OPCs, proteins associated with TET1 were involved in cell homeostasis or protein synthesis. Oligodendrocytes display a strict vesicular transport system including protein folding, protein sorting, formation of carrier vesicles, vesicle transport along elements of the cytoskeleton, and vesicle targeting/fusion [26]. The synchronization and coordinate of vesicle transport are essential to maintain the structural and functional organization of oligodendrocytes. In addition, many genes closely related to neurological diseases, such as Got1, ApoE, Gm2a, have been found to interact with TET1 in OPCs [27–30]; and Cst3 is associated with dementia in Lewy body disease (24) and Alzheimer's Disease (25). Different from OPCs, PPI networks for TET1 are relatively simple in OL, including ATP activity, nuclear pore outer ring and regulation of DNA metabolic process. These TET1-associated terms suggest unknown functional settings for TET1 in OLs as a supplement to the GO functional annotation analyses. Together, our results provide novel perspectives into distinctive functions beyond transcriptional regulation role for TET1 in oligodendrocyte biology.

Although there are no reports regarding the involvement of TET1 in most of above biological processes, some studies can explain our observations to some extent. For example, one of the TET1-IP products in OPC, Calpain, a protein belonging to the family of calcium-dependent, non-lysosomal cysteine proteases, could mediate TET1 degradation in mouse embryonic stem cells (ESCs) [31]. Studies have suggested putative involvement of TETs in the formation of 5hmC in mitochondria DNA (mtDNA), which is consistent with the mitochondria associated proteins (e.g. Abcb6, Acly) in TET1-IP products from OPC cultures. In purified cerebellum granule neuron cultures, TET1 and TET2 presence not only in the nucleus but also in the mitochondrial fraction identified by Western Blot assay [32]; mouse 3T3-L1 cells treated with histone deacetylase inhibitor show reduced 5hmC content in mtDNA and decreased mitochondrial TET1 expression [33]. We anticipate that future studies extending the role of TETs beyond genomic DNA, i.e., into the field of mitochondrial epigenetics, will likewise reveal functional diversity for TET family proteins in the central nervous system.

Regarding the transcriptional functions, interacting partners of TETs may also contribute to their recruitment to specific genomic regions. In mouse ESCs, the pluripotency factor NANOG physically interacts with TET1, and NANOG depletion results in reduced TET1 binding at NANOG-bound regions [34]. Similarly, PR domain zinc finger protein 14 (PRDM14) [35], Polycomb repressive complex 2 (PRC2) [36]and LIN28A [37] have also been reported to interact with and recruit TET proteins in mouse ESCs. TET1 could promote glycosylation of chromatin by binding to O-N acetyl glucose transferase (OGT) and mediate posttranscriptional modification [38]. A recent study indicates that EGR1 interacts and recruits TET1 to its target binding sites [39]. Collectively, these results imply that the interacting partners of TETs, in many cases key transcription factors of the cells studied, contribute to TETs recruitment into target genes. Further analysis is needed to determine whether the interaction per se mediates the recruitment or instead the interacting partner helps to establish a favorable chromatin environment for TET binding of DNA.

TET proteins are iron (II)/α-ketoglutarate (Fe (II)/α-KG)-dependent dioxygenases. The core catalytic domain at the carboxyl terminus is comprised of a double-stranded β-helix (DSBH) domain and a cysteine-rich domain [40]. Full-length TET1 have a CXXC zinc finger DNA binding domain at amino terminus; however, the CXXC domain of TET1 has no DNA binding activity and is dispensable for its catalytic activity in vivo [41]. This implies that other proteins are involved in DNA binding of TET1, a necessary step to promote the conversion of 5-mC to 5-hmC. Interestingly, mouse TET1 preferentially exists in an N-terminus-truncated form (known as TET1s) in somatic tissues but exists in its full-length form (known as TET1e) in early embryos [40]. TET1s, which does not have a CXXC domain and the other N-terminal sequence, has reduced global chromatin binding compared with TET1e and confers weaker demethylation activity in cells. Therefore, it is important to further investigate the function and mechanism of individual forms of TET1 in different cell types.

In our study, both Olig2 and HDAC1 were shown to interact with TET1 in oligodendrocytes. HDAC1 has been identified to be recruited specifically by TET1 in male germline stem cells [23] and this complex binds to key genes to regulated histone acetylation and gene expression. Therefore, we speculate that in oligodendrocytes, TET1 may play a role of recruitment with HDAC1 to affect histone acetylation, which may further influence chromosome structure and gene transcription activation. As one of the OL lineage specific TFs, Olig2 belongs to the basic helix-loop-helix (bHLH) transcription factor family and is necessary for oligodendrocyte development [42]. All bHLH transcription factors function in a dimeric state as homodimers or as heterodimers with another bHLH protein. Once in contact with the promoter or enhancer elements of a target, bHLH homodimers and heterodimers serve as scaffolding upon which a multimeric complex of transcriptional co-regulator proteins can be assembled. Olig2 has been shown to interact with NKX2.2 [43] and histone acetyl transferase p300 [44], all suggesting the transcriptional activator role of Olig2 in OL development. Our identification of TET1 as novel Olig2 co-factor thus provide further clue for Olig2 function in modulating oligodendrocytes development.

Overall, the comprehensive analysis of endogenous TET1 interactome highlights many novel partners with interesting roles and provide a basis for further functional investigations of TET1 in oligodendrocytes biology and related disease.

## Data Availability

All data generated or analyzed during this study are included in this published article.
